# The influence of adjuvant chemotherapy dose intensity on overall survival in resected colon cancer: a multicentered retrospective analysis

**DOI:** 10.1186/s12885-022-10198-y

**Published:** 2022-11-01

**Authors:** Daniel Breadner, Jonathan M. Loree, Winson Y. Cheung, Meghan Gipson, Suganija Lakkunarajah, Karen E. Mulder, Jennifer L. Spartlin, Shiying Kong, Philip Q. Ding, Sharlene Gill, Stephen A. Welch

**Affiliations:** 1 Department of Oncology, A3-924 LRCP Medical Oncology, London Regional Cancer Program, 800 Commissioners Road East, London, ON N6A5W9 Canada; 2 Schulich School of Medicine and Dentistry at, Western University, London, ON Canada; 3 BC Cancer, Vancouver, BC Canada; 4 Department of Oncology, Arnie Charbonneau Cancer Institute, Calgary, AB Canada; 5 Department of Medicine, Royal College of Surgeons in Ireland, Dublin, Ireland; 6 Department of Oncology, Cross Cancer Institute, Edmonton, AB Canada; 7 Oncology Outcomes, Calgary, AB Canada; 8 Faculty of Medicine & Dentistry, University of Alberta, Edmonton, AB Canada

**Keywords:** Adjuvant colon cancer, Dose intensity, Relative dose intensity, Oxaliplatin, Toxicity

## Abstract

**Background:**

Colorectal cancer remains the second leading cause of cancer death in North America. Fluorouracil and oxaliplatin based adjuvant chemotherapy for resected colon cancer (CC) reduces cancer recurrence, but also causes significant toxicity requiring dose reductions. The effect of dose intensity on survival outcomes is not fully understood and strengthening the evidence supports informed decision making between patients and oncologists.

**Methods:**

Patients treated with adjuvant chemotherapy, between 2006 and 2011, for resected colon cancer at four Canadian academic cancer centers were retrospectively analyzed. All patients must have received oxaliplatin with either capecitabine (CAPOX) or 5-FU (FOLFOX). Dose intensity (DI) was calculated as total delivered dose of an individual chemotherapy agent divided by the cumulative intended dose of that agent. The influence of DI on overall survival was examined.

**Results:**

Five hundred thirty-one patients with high-risk stage II or stage III resected CC were eligible and included in the analysis. FOLFOX was the most common regimen (69.6%) with 29.7% of patients receiving CAPOX and 0.7% receiving both therapies. Median follow-up was 36.7 months. The median DI for 5-FU and capecitabine was 100% and 100% with 13.6% and 9.8% of patients receiving ≤ 80% DI, respectively. The median DI of oxaliplatin was 70% with 56.8% of patients receiving ≤ 80% DI. A DI of > 80% for each chemotherapy component was associated with a significant improvement in overall survival compared to those with a DI of ≤ 80% (5-FU HR = 0.23, 95% CI = 0.08–0.65, *p* = 0.006; capecitabine HR = 0.56, 95% CI = 0.33–0.94, *p* = 0.026; oxaliplatin HR = 0.52, 95% CI = 0.33–0.82, *p* = 0.005). Patients with T2 and/or N2 disease with an oxaliplatin DI > 80% had a trend towards improved survival (HR = 0.62, 95% CI = 0.38–1.02, *p* = 0.06).

**Conclusions:**

In resected CC an adjuvant chemotherapy DI of > 80%, of each chemotherapy agent, is associated with improved overall survival.

## Intro

In the year 2021, it is estimated that 24,800 Canadians will be diagnosed with and 9,600 will die from colorectal cancer (CRC) accounting for 11% of cancers diagnoses and 12% of cancer-related deaths [1]. Recently, rates of CRC cases and deaths have been decreasing due to increased screening, however CRC remains the third leading cause of cancer and second leading cause of cancer-related death in Canada [2].

For most of the last two decades, six months of treatment with adjuvant FOLFOX (5-florouracil with oxaliplatin and leucovorin) or CAPOX (capecitabine and oxaliplatin) were standard for treatment of stage III colon cancer (CC), after curative resection [3]. Since 2006, many studies have contributed to the changes in adjuvant therapy for stage III CC. These changes include the resection of 12 or more lymph nodes from the tumour site and the addition of oxaliplatin to 5-FU [4, 5]. Dosages and the delivery of 5-FU have also been altered so that they are more tolerable. However, the peripheral sensory neuropathy caused by oxaliplatin can have long term, life-altering effects on patients and often leads to dose modifications which have the potential to impact patient outcomes [4–7].

A dose intensity (DI) of over 70–80% has been associated with increased response rate, recurrence free survival, progression free survival, and overall survival (OS) in both the adjuvant and metastatic setting across numerous cancer types, including CC [5, 8, 9]. Exploration of the impact of DI on clinical outcomes in CC provides data to allow for better informed treatment decisions by oncologists and their patients. The current paper examines the effect of DI on OS for patients with high risk resected stage II and stage III colon cancer receiving doublet chemotherapy with adjuvant FOLFOX or CAPOX.

## Methods

### Population and data collection

Patients from four Canadian academic cancer centers with high-risk stage II or stage III resected adenocarcinoma of the colon that received adjuvant chemotherapy with oxaliplatin and a fluoropyrimidine between 2006 and 2011 were included in the analysis. Those with rectal cancers, those who received any neoadjuvant therapy, positive surgical margins, had known residual disease, and those with any metastatic lesions (resected or in situ) were excluded from the analysis. Patients with concurrent or recent malignancy, other than non-melanoma skin cancer, were excluded. Data sharing agreements and institutional ethics approval were obtained. This retrospective study was conducted in accordance with the relevant guidelines and regulations. Patient data was acquired with the approval of the Institutional Review Board of Western University (REB#102,843).

### Study procedures

Tumor-node-metastasis (TNM) staging was based on the American Joint Committee on Cancer staging manual, seventh edition. Chemotherapy regimens include CAPOX comprised of oxaliplatin 130 mg/m^2^ IV and capecitabine 1000 mg/m^2^ orally twice daily for day 1–14, delivered every 21 days for 8 cycles and modified FOLFOX6 (FOLFOX) with oxaliplatin 85 mg/m^2^ IV, leucovorin 400 mg/m^2^ IV, 5-FU bolus 400 mg/m^2^ IV then 5-FU infusion 2400 mg/m^2^ IV over 46 h, delivered once every 14 days for 12 cycles. DI was calculated as total delivered dose of an individual chemotherapy agent divided by the cumulative intended dose of that agent. In FOLFOX the dose of bolus and infusional 5-FU are combined.

### Statistical analysis

Demographic and clinical characteristics were analyzed using descriptive statistics. Multivariable analysis was performed by building Cox proportional hazards models to identify associations between chemotherapy DI and patient OS, adjusting for age and TNM classification for tumor and nodal staging. Survival analyses were performed using the Kaplan–Meier method with comparisons using the log-rank test. An alpha level of 0.05 was used for all analyses.

## Results

### Patient characteristics

Between 2006 and 2011, 531 patients with resected high-risk stage 2 and stage 3 CC were treated at the 4 participating academic centres across Canada (Vancouver, Edmonton, Calgary, and London). 526 of these patients were included in this study. All patients received an oxaliplatin-based therapy (either CAPOX or FOLFOX) with 69.6% (*n* = 366) of patients receiving 5-FU and 29.7% (*n* = 156) of patients receiving capecitabine, and 0.7% (*n* = 4) of patients received a mix of CAPOX and FOLFOX. Baseline characteristics are shown in Table 1. Median age was 62 years and median length of follow-up was 36.7 months. The median DI for capecitabine and 5-FU was 100%, with 9.8% of patients and 13.6% of patients receiving ≤ 80% DI, respectively. The median DI for oxaliplatin was 70%, with 56.8% of patients receiving ≤ 80% DI. A larger proportion of females, compared to males, received ≤ 80% DI of oxaliplatin (66.1% vs. 51.4%). Baseline characteristics were balanced between those receiving CAPOX versus FOLFOX, Table 1.

**Table 1:** Baseline characteristics

	*n* = 531	CAPOX *n* = 156	FOLFOX *n* = 366	Oxaliplatin DI > 80% *n* = 221	Oxaliplatin DI ≤ 80% *n* = 310
Age -median (IGR)	62 (54 – 68)	63 (55 – 68)	60 (50 – 67)	61 (55 – 68)	62 (53 – 68)
Age -mean (STD)	60.3 (10.5)	58.5 (10.8)	61 (10.3)	60.4 (9.7)	60.1 (11)
Age -range	19 – 83	24 – 79	19 – 83	24 – 77	19 – 83
Sex – Female	251 (47.3%)	65 (41.7%)	179 (48.9%)	85 (33.9%)	166 (66.1%)
Sex – Male	280 (52.7%)	91 (58.3%)	187 (51.1%)	136 (48.6%)	144 (51.4%)
Median Follow-up (IQR) days	1100 (937 – 1551)	1030 (870.5—1104)	1217 (973—1791)	1168 (918—1552)	1083 (960—1540)
T0-2	60 (11.3%)	10.9%	11.5%	13.7%	9.7%
T3	301 (56.7%)	57.1%	56.6%	54.3%	58.4%
T4	166 (31.3%)	31.4%	31.1%	31.7%	31%
N0	28 (5.3%)	3.2%	5.2%	1.8%	7.7%
N1	314 (59.1%)	56.4%	60.7%	62%	57.1%
N2	187 (35.2%)	40.4%	33.6%	35.7%	34.8%
Deceased	18.1% (96 events)	18 (11.5%)	75 (20.5%)	29 (13.1%)	67 (21.6%)

### Overall survival with dose intensity

**Fig. 1:**
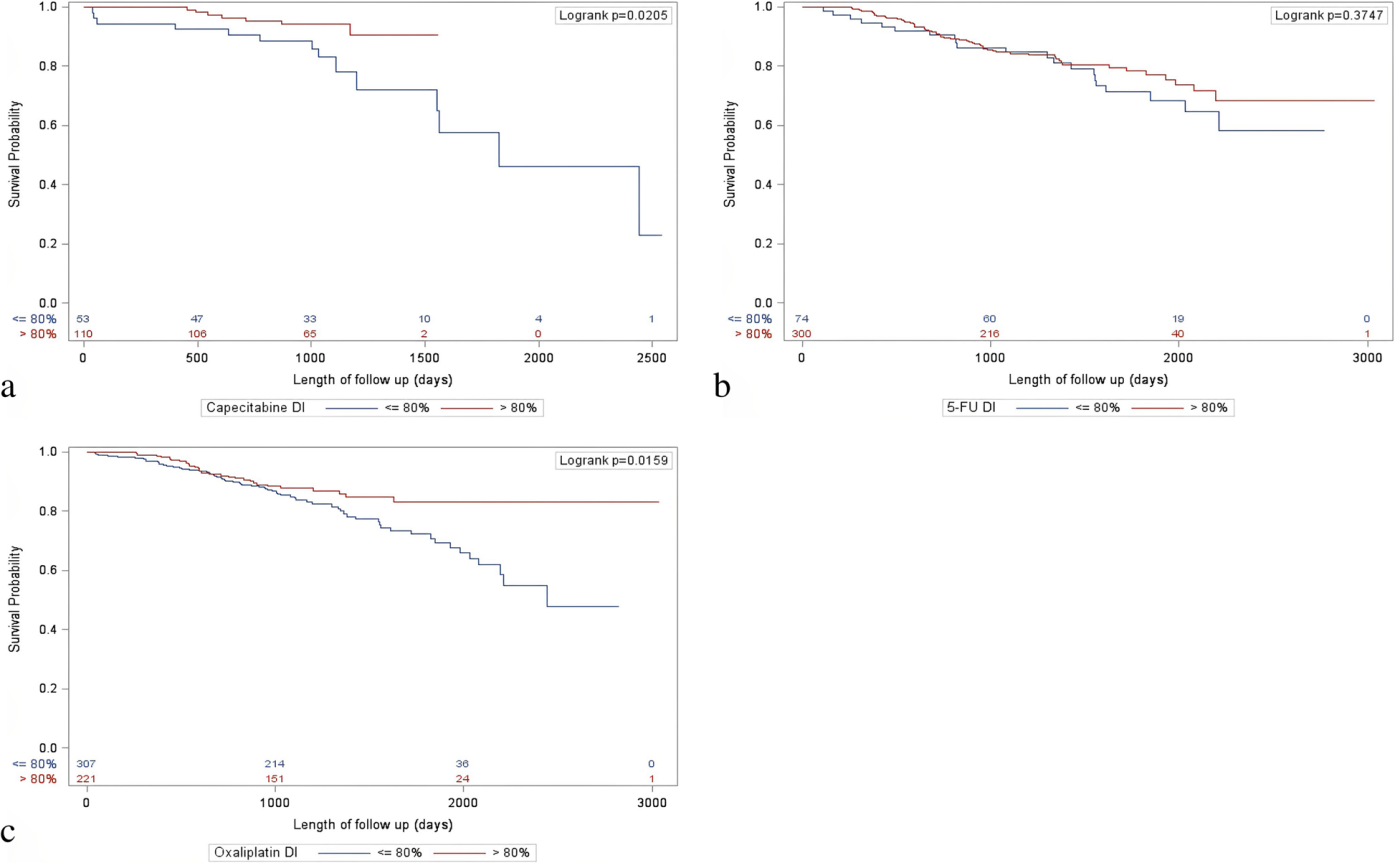
Kaplan–Meier survival curves demonstrating the effect of dose greater than 80% versus less than or equal to 80% for each chemotherapy component **a** capecitabine **b** 5-FU and **c** oxaliplatin

Kaplan–Meier plots for OS stratified by DI (≤ 80% vs. > 80%) of capecitabine, 5-FU, and oxaliplatin are shown in Fig. 1a-c. In the multivariate analysis of OS based on DI, Table 2, the patients who received > 80% DI of the planned 5-FU had a survival benefit (HR = 0.56; 95% CI = 0.33–0.94, *p* = 0.029) when compared to those with DI ≤ 80%. The patients who received > 80% DI of capecitabine had significantly improved OS (HR = 0.23; 95% CI = 0.08–0.65, *p* = 0.006) compared with those with DI ≤ 80%. Patients who received > 80% DI of oxaliplatin also had an improvement in OS (HR = 0.52; 95% CI = 0.33–0.82, *p* = 0.005). Death was a more common outcome in patients receiving ≤ 80% DI of either doublet therapy: 21.6% in ≤ 80% oxaliplatin compared to 13.1% in > 80% DI; 26.4% in ≤ 80% DI capecitabine compared to 6.3% in > 80% DI; 28.4% in ≤ 80% DI 5-FU compared to 18.9% in > 80% DI, Table 3.

**Table 2:** Multivariable analyses of associations between patient characteristics and overall survival

Characteristic	Category	FOLFOX (5-FU dose intensity) (*n* = 371)		CAPOX (Capecitabine dose intensity) (*n* = 164)		All patients (Oxaliplatin dose intensity) (*n* = 526)	
		**Hazard Ratio (95% Confidence Interval)**	***P* value**	**Hazard Ratio (95% Confidence Interval)**	***P* value**	**Hazard Ratio (95% Confidence Interval)**	***P* value**
Age at chemo start		0.99 (0.97 to 1.02)	0.62	1.01 (0.96 to 1.06)	0.70	1.00 (0.98 to 1.02)	0.95
Sex	Female	Reference	0.59	Reference	0.18	Reference	0.46
	Male	1.13 (0.71 to 1.80)		2.05 (0.73 to 5.77)		1.17 (0.77 to 1.77)	
Dose intensity	≤ 80%	Reference	0.03	Reference	0.006	Reference	0.005
	> 80%	0.56 (0.33 to 0.94)		0.23 (0.08 to 0.65)		0.52 (0.33 to 0.82)	
T stage	T0-T2	Reference	0.03	–	–	Reference	< 0.001
	T3	1.47 (0.58 to 3.75)		–	–	1.66 (0.65 to 4.19)	
	T4	2.56 (1 to 6.6)		–	–	3.33 (1.32 to 8.41)	
N stage	N0	Reference	< 0.001	Reference	0.03	Reference	< 0.001
	N1	7.20 (0.96 to 53.72)		1.44 (0.16 to 13.05)		7.55 (1.03 to 55.51)	
	N2	20.70 (2.79 to 153.3)		4.70 (0.57 to 38.85)		19.72 (2.71 to 143.54)

**Table 3:** Deaths by chemotherapy component and dose intensity

	Capecitabine DI > 80% *n* = 112	Capecitabine DI ≤ 80% *n* = 53	5-FU DI > 80% *n* = 301	5-FU DI ≤ 80% *n* = 74	Oxaliplatin DI > 80% *n* = 221	Oxaliplatin DI ≤ 80% *n* = 310
Deaths	7 (6.3%)	14 (26.4%)	57 (18.9%)	21 (28.4%)	29 (13.1%)	67 (21.6%)

### Overall survival with tumour and nodal staging

In the multivariate analysis of OS based on tumour and nodal staging for all patients, right column in Table 2, patients with T4 disease, compared to T0-T2 disease had an increased risk of death (HR = 3.33; 95% CI = 1.32–8.41, *p* = 0.011), while T3 disease was not significantly associated with an increased risk of death (HR 1.66; 95% CI = 0.65–4.19, *p* = 0.287). Patients with both N1 and N2 disease had an increased risk of death, compared to those with N0 disease, (N1: HR = 7.55; 95% CI = 1.03–55.51, *p* = 0.047 and N2: HR = 19.72; 95% CI = 2.71–143.54, *p* = 0.003).

The multivariate analysis for OS in all patients with T4 and/or N2 disease (*n* = 286) receiving > 80% DI of oxaliplatin compared to ≤ 80% DI of oxaliplatin had a trend towards improved survival although it did not reach significance, HR = 0.62; 95% CI = 0.38–1.02, *p* = 0.06.

## Discussion

This multicentre retrospective analysis showed that there was a statistically significant improvement in the OS for patients receiving > 80% DI of their oxaliplatin therapy. There was also a significant improvement in OS derived from either 5-FU or capecitabine, as a component of doublet therapy, when patients had a DI of > 80%. This study also confirms patients with T4 and/or N2 disease are at higher risk of early death, compared to those with earlier T or N stage disease. Lastly, in patients with T4 or N2 disease, this study showed a trend for improved survival in those patients with an oxaliplatin DI of > 80% compared to those with an oxaliplatin DI of ≤ 80%. As a result, this study confirms that higher DI is correlated with increased OS [10].

This study calculated DI by comparing the dose received to the dose suggested for the regimen, a formula based on findings by Hryniuk [11]. The time taken to complete treatment is not accounted for in this definition, but would be captured in some relative dose intensity calculations, as a result, this analysis cannot assess the effect of dose delays on patient outcomes.

Reductions in dose and schedule modifications are common for real-world patients receiving chemotherapy due to the incidence of adverse events [12]. Oxaliplatin induced neuropathy, which can leave patients with permanent morbidity, is a feared toxicity which has led to studies attempting to reduce the duration of oxaliplatin or mitigate its risk [13]. As a result, balancing effective dosages of chemotherapy with side effects is an essential part of cancer treatment. However, higher DI is correlated with increased OS in adjuvant CC as well as other cancer treatment settings [5, 8, 10, 12, 14]. This study confirms that DI of > 80% is correlated with better OS for patients receiving CAPOX or FOLFOX when compared to patients receiving less than 80% of their intended therapy. Our study includes a diverse group of patients from 4 academic centers across Canada, and since it was not focused on a particular demographic, we believe the findings to be broadly applicable to patients with high risk stage II and III CC patients.

The patients included in this analysis were treated before the IDEA collaboration reported that 3 months of CAPOX therapy was non-inferior to the 6 month regimen in patients without T4 or N2 disease [3]. In patients treated with 3 months of therapy, there is also a reduction in toxicity and adverse effects [3, 15]. Therefore, this analysis used the intended dose of CAPOX or FOLFOX over 6 months to determine the DI. The subgroup of patients with T4 and/or N2 disease demonstrated a trend towards improved survival when DI was > 80%, consistent with findings of this study and the results of the IDEA collaboration, supporting that 6 months of adjuvant therapy should remain the goal for this population.

As is the nature of retrospective analyses, this study has some potential limitations and can only show association, not causation. This study examined all-cause mortality, which means patients with comorbid diseases or early recurrence during adjuvant chemotherapy would bias the findings. Cancer associated mortality would be a more relevant endpoint to account for comorbid disease. An assessment taking into consideration not only DI but also the time between resection of the CC and commencing chemotherapy and the effect of dose delays during adjuvant therapy would better assess risk factors for recurrent disease and death [16]. Additionally, since this study was completed before the IDEA collaboration results were released, it would be useful to analyse the effect of DI during the first 3 months of adjuvant therapy, or based on the desired treatment duration.

## Conclusion

In this multicentre retrospective analysis, there was a statistically significant improvement in the overall survival in those receiving a DI of > 80% of each individual chemotherapy drug for patients receiving adjuvant treatment for CC. There is a trend towards improved survival for patients with T4 and/or N2 disease receiving at least 80% of the intended oxaliplatin. This information can contribute to informed discussions between patients and clinicians when assessing the need for dose reductions during adjuvant chemotherapy for resected colon cancer.

## Data Availability

The datasets used and/or analysed during the current study are available from the corresponding author on reasonable request.
